# Simultaneous determination of multiple components in rat plasma by UHPLC-sMRM for pharmacokinetic studies after oral administration of Qingjin Yiqi Granules

**DOI:** 10.3389/fphar.2023.1155973

**Published:** 2023-04-13

**Authors:** Xiaohua Yang, Shujing Chen, Kunze Du, Ye Shang, Shiming Fang, Jin Li, Han Zhang, Yanxu Chang

**Affiliations:** ^1^ State Key Laboratory of Component-based Chinese Medicine, Tianjin University of Traditional Chinese Medicine, Tianjin, China; ^2^ Tianjin Key Laboratory of Phytochemistry and Pharmaceutical Analysis, Tianjin University of Traditional Chinese Medicine, Tianjin, China; ^3^ Haihe Laboratory of Modern Chinese Medicine, Tianjin, China

**Keywords:** Qingjin Yiqi Granules, Pharmacokinetics, COVID-19, UHPLC-sMRM, Traditional Chinese Medicine

## Abstract

As a Traditional Chinese Medicine prescription, Qingjin Yiqi Granules (QJYQ) provides an effective treatment for patients recovering from COVID-19. However, the pharmacokinetics characteristics of the main components of QJYQ *in vivo* are still unknown. An efficacious ultra-performance liquid chromatography-tandem mass spectrometry (UHPLC-MS/MS) was developed and validated for the simultaneous determination of 33 components in rat plasma after oral administration of QJYQ. The plasma samples were precipitated with 400 µL methanol/acetonitrile (1/1, *v*/*v*) and analyzed in scheduled multiple reaction monitoring mode. The linear relationship of the 33 components was good (r > 0.9928). The lower limit of quantification for 33 components ranged from 0.4–60.5 ng/mL. The average recoveries and matrix effects of the analytes ranged from 72.9% to 115.0% with RSD of 1.4%–15.0%. All inter-day and intra-day RSDs were within 15.0%. After oral administration (3.15 g/kg), the validated approach was effectively applied to the pharmacokinetics of main components of QJYQ. Finally, fifteen main constituents of QJYQ with large plasma exposure were obtained, including baicalin, wogonoside, wogonin, apigenin-7-*O*-glucuronide, verbenalin, isoferulic acid, hesperidin, liquiritin, harpagide, protocatechuic acid, *p*-Coumaric acid, ferulic acid, sinapic acid, liquiritin apioside and glycyrrhizic acid. The present research lays a foundation for clarifying the therapeutic material basis of QJYQ and provides a reference for further scientific research and clinical application of QJYQ.

## 1 Introduction

Traditional Chinese medicines (TCMs) have been used clinically for thousands of years as natural healing agents. TCMs treatment has the advantages of fewer side effects and low toxicity. It can play a more comprehensive role in the treatment of diseases through its unique multi-target (Xiang, et al., 2021; Duya, et al., 2022; Li, et al., 2023). It has a special function in treating tough and complex disorders in particular, and it is indispensable in chemical medicine (Luo, et al., 2020; Zhang, et al., 2020; Liao, et al., 2022).

Pharmacokinetic study of TCMs components is an important bridge between the study of chemical composition and active components of TCMs, mainly to elucidate the absorption, distribution, metabolism and excretion characteristics of various major chemical components *in vivo* (Lu et al., 2008; Huang, et al., 2022). Pharmacokinetic study can identify components that have significant systemic exposure in the systemic blood after administration (Liu et al., 2009). This provides the key research object for the material basis research of the curative effect of TCMs.

Due to the complexity and huge difference in content of the ingredients contained in TCMs, it is difficult to analyze and determine multiple components *in vivo.* Therefore, the development of sensitive and reliable biological sample analysis for simultaneous quantitative determination of multiple components *in vivo* is a focus of the study of pharmacokinetics of multiple components in TCMs (Zou, et al., 2019). Ultra-performance liquid chromatography-tandem mass spectrometry (UHPLC-MS/MS) is often used for the analysis of complex components of TCMs. The schedule multiple reaction monitoring mode (sMRM) can solve the problem that the peak is too thin and the scanning points are not enough, compared with MRM mode. This acquisition mode greatly improves the efficiency of quantitative analysis (Li et al., 2021).

Qingjin Yiqi Granules (QJYQ) was developed by Academician Zhang Boli, which is used to treat the body damage and immune system adjustment for patients recovering from COVID-19. QJYQ consists of *Ginseng radix et rhizoma, Ophiopogonis radix, Schisandrae chinensis fructus* of principle medicine*, Poria, Pinelliae rhizoma praeparatum cum alumine*, bran stir-baked *Atractylodis Rhizoma*, *Citri reticulatae pericarpium, Coicis semen* of minister medicines*, Scrophulariae radix, Cimicifugae rhizoma, Bupleuri radix, Scutellariae radix, Phragmitis rhizoma, Lophatheri herba* of assistant medicine*, verbenae herba, Glycyrrhizae radix et rhizoma* of envoy medicines. The detailed components of QJYQ are presented in the literature (Yang, et al., 2023). These 16 Chinese medicines mostly contain phenolic acids, flavonoids, iridoids and triterpenoid saponins (Tarnawski et al., 2006; Lara-Issasi et al., 2019). At present, QJYQ has been widely used as a rehabilitation drug for discharged patients with COVID-19 in Hebei and Tianjin of China. QJYQ can effectively treat low fever during the recovery period of COVID-19 (Wang, et al., 2021; Tian, et al., 2022). In addition, QJYQ can improve symptoms of breathlessness and fatigue in convalescent patients (Pang, et al., 2022). However, there are no publications about the pharmacokinetic of multiple components in rat plasma after oral administration of QJYQ.

In this study, an UHPLC-sMRM method was established for simultaneous determination of thirty-three compounds in rat plasma to explore the main absorbed compounds of QJYQ. Moreover, a total of 15 main compounds with large plasma exposure in rat plasma were detected, including baicalin, wogonoside, wogonin, apigenin-7-*O-*glucuronide, verbenalin, isoferulic acid, sinapic acid, hesperidin, *p*-Coumaric acid, glycyrrhizic acid, liquiritin, ferulic acid, harpagide, protocatechuic acid and liquiritin apioside. This study provides comprehensive insights into the pharmacokinetic of QJYQ, and would be valuable for future clinical development and utilization of QJYQ.

## 2 Materials and methods

### 2.1 Chemicals and reagents

Acetonitrile and methanol were HPLC-grade from Fisher Scientific (Pittsburg, PA, United States). All other reagents were of analytical grade and obtained by Anaqua™ Chemicals Supply (Wilmington, DE, United States). Ultrapure water is prepared by the Millipore Ultra-Pure Water System. Harpagide, protocatechuic acid, atractyloside A, verbenalin, paeoniflorin, *p*-Coumaric acid, sinapic acid, vitexin, liquiritin, liquiritin apioside, isoliquiritin apioside, cimifugin, scutellarin, apigenin-7-*O*-glucuronide, ononin, isoliquiritin, naringenin, glycyrrhizic acid, baicalein, icariin (IS, internal standards), isopimpinellin (IS), astragaloside II (IS) were purchased from Chengdu Desite Bio-Technology Co., Ltd (Chengdu, China). Quercitrin were purchased from Shanghai yuanye Bio-Technology Co., Ltd. (Shanghai, China). Catechin, cryptochlorogenin acid, hyperoside, wogonoside, chlorogenic acid, ginsenosides Rf, ginsenosides Rh1 were purchased from Chengdu Must Bio-Technology Co., Ltd. (Chengdu, China). Isoferulic acid, hesperidin, baicalin, harpagoside, wogonin, ferulic acid were purchased from National Institutes for Food and Drug Control (Beijing, China). Their purity was higher than 98%. QJYQ were made by the laboratory of Tianjin University of Traditional Chinese Medicine, and the batch numbers in this study was 210601.

### 2.2 UHPLC-sMRM conditions

Exion LC AD tandem Blueline 3500 triple quadrupole mass spectrometer (AB SCIEX, Framingham, MA, United States) was used to thirty-three compounds in rat plasma. The conditions of liquid chromatography and mass spectrometry are the same as those for the quantitative determination of 50 components *in vitro* by UHPLC-sMRM (Yang, et al., 2023). Briefly, quantitation was operated using sMRM of the transitions of m/z 362.9→138.9 for harpagide at 2.10 min, m/z 152.9→109.0 for protocatechuic acid at 2.40 min, m/z 433.0→225.0 for verbenalin at 5.56 min, m/z 163.0→119.0 for *p*-Coumaric acid at 6.32 min, m/z 192.9→134.0 for ferulic acid at 7.08 min, m/z 222.9→164.0 for sinapic acid at 7.26 min, m/z 417.0→134.8 for liquiritin at 7.32 min, m/z 549.1→255.0 for liquiritin apioside at 7.43 min, m/z 193.0→133.0 for isoferulic acid at 7.48 min, m/z 609.0→301.0 for hesperidin at 9.12 min, m/z 445.1→268.8 for apigenin-7-*O*-glucuronide at 9.25 min, m/z 445.0→268.9 for baicalin at 10.90 min, m/z 459.0→268.0 for wogonoside at 13.62 min, m/z 821.1→351.1 for glycyrrhizic acid at 17.56 min and m/z 282.8→267.9 for wogonin at 17.91 min. The whole detailed parameters of sMRM were obtained in Supplementary Table S1.

### 2.3 Preparation of standard and quality control (QC) samples

Baicalin and wogonoside were accurately weighed and dissolved in methanol to obtain the standard stock solutions at the concentration of 2 mg/mL. The other 31 compounds were prepared into 1 mg/mL in methanol. The ISs (icariin, isopimpinellin and astragaloside II) were configured in the same way as 1 μg/mL. The mixed stock solution was stepwise diluted to desired concentrations with methanol for plotting standard curves. Each mixed standard solution was accurately taken and diluted with methanol to prepare 4 different concentrations of lower limit of quantification (LLOQ), low, medium and high as QC reserve solution. All solutions are stored in the refrigerator at 4°C for future use.

### 2.4 Preparation of plasma sample

Rat plasma (100 μL) was mixed with 10% formic acid (10 μL) and vortexed for 1 min at room temperature. ISs (10 μL) and methanol/acetonitrile (1/1, *v/v*) (400 μL) were added and vortexed for 5 min, then centrifuged for 10 min at 14,000 rpm and 4°C. The transported supernatant was condensed to dryness under the flow of nitrogen gas. The dried residue was reconstituted with 70% methanol (100 μL) by vortex-mixing for 5 min and centrifuging at 14,000 rpm for 10 min. Last, 2 μL of the solution were injected into the UHPLC-sMRM system.

### 2.5 Method validation

The UHPLC-sMRM bioanalytical method was validated for specificity, LLOQ, linearity, accuracy and precision, extraction recovery, matrix effects and stability. The results should comply with the currently recognized U.S. Food and Drug Administration (FDA) bioanalytical method validation guidelines.

#### 2.5.1 Selectivity

The selectivity was determined by comparing the chromatograms of six separate batches of blank rat plasma samples, blank plasma spiked with corresponding mixed standards and ISs, and real plasma samples obtained following oral administration of QJYQ.

#### 2.5.2 Linearity and LLOQ

Linearity investigation was conducted by adding a mixed solution of reference substances with 8 concentration levels into blank plasma, processing the samples according to the preparation method of plasma samples, and sampling analysis. The ratio of the analyte peak area to the ISs peak area was taken as the vertical coordinate (y), and the concentration of analyte was taken as the horizontal coordinate (x). The weighted (1/X, 1/X^2^) least squares linear regression was used to establish the linear relationship. The LLOQ was determined by analyzing blank plasma spiked with mixed standards at a signal to noise ratio of approximately 10.

#### 2.5.3 Precision and accuracy

QC at four concentrations were added to blank plasma (n = 6), and the samples were treated according to the plasma sample preparation method. The intra-day precision and accuracy were evaluated by analyzing samples within the same day. The inter-day precision and accuracy were verified by repeating the same procedure for three consecutive days and applying the accompanying standard curve.

#### 2.5.4 The recovery and matrix effect

The extraction recovery and matrix effect of six replicate groups were determined at 4 QC levels. The recovery is determined by comparing the peak area of the analyte in the pre-extraction spiked plasma samples with the spiked solutions in the post-extraction blank plasma. The matrix effect is tested by comparing the peak area of the post-extracted spiked samples with that of the standard solution of four different concentrations in six replicates of QC samples. A single concentration of ISs was also determined by the above method.

#### 2.5.5 Stability

The stability of plasma samples needs to be evaluated based on processing and storage conditions. The stability of analytes in plasma was evaluated by analyzing 4 QC samples (n = 6). The stability was included auto-sampler stability (keeping the sample in auto-sampler for 24 h), three times of freeze-thaw cycles stability (freezing cycle at −80°C, thawing cycle at room temperature), room temperature stability (storing samples at room temperature for 24 h), long-term stability (storing samples at −80°C for 1 month). In addition, the stability of the working solution needs to be determined (the working solution is stored at 4°C for 1 month). Accompanying curve was established when testing the sample, calculate and compare the ratio of the actual measured concentration to the theoretical concentration, expressed in accuracy and RSD.

### 2.6 Application for pharmacokinetic study

The pharmacokinetic study was approved by the Animal Ethics Committee of Tianjin University of Traditional Chinese Medicine (TCM-LAEC2021228). Ten male Sprague-Dawley rats (weight 220–240 g) was kept at the animal center of Tianjin University of Traditional Chinese Medicine (Tianjin, China). Rats were fasted for 12 h and drank water freely before administration. According to the clinical dose, the dose in rat is 3.15 g/kg. Blood samples (about 100 μL) were collected before dosing and at 0.083, 0.17, 0.25, 0.33, 0.5,0.75, 1, 2, 3, 4, 6, 8, 10, 12, 24, 36 h after administration of QJYQ from vein of the eye sockets into heparinized tubes. The sample was transferred right away following a 10 min period of centrifugation at 7000 rpm and 4°C. All plasma samples were frozen and stored at −80°C.

### 2.7 Data analysis for pharmacokinetic study

All the pharmacokinetic parameters were calculated using the DAS 1.0 software (Drug and Statistics 1.0, Medical College of Wannan, China). Pharmacokinetic parameters include the maximum drug concentration in plasma (C_max_), the time to achieve maximum drug concentration (T_max_), the area under the plasma concentration-time curve (AUC), elimination half-life (T_1/2_), and mean residence time (MRT). The pharmacokinetic parameters were computed using the non-compartmental model. All data are expressed as mean ± standard deviation (SD). GraphPad Prism 8.0 (GraphPad Software Inc., La Jolla, CA, United States) software was used to draw the drug-time curve.

## 3 Results

### 3.1 Optimization of UHPLC-sMRM conditions

The liquid chromatography conditions were optimized to obtain better separation in a short time. Different mobile phases (acetonitrile-water, methanol-water), concentrations of additive (0.05%, 0.1%, and 0.2% formic acid), column temperatures (30, 35°C and 40°C) and flow rates (0.2, 0.3 and 0.4 mL/min) were optimized. The results show that a good separation effect and stable and high response value can be achieved under the following conditions: acetonitrile-0.1% formic acid in water as mobile phases, flow rate at 0.3 mL/min and column temperature at 40°C (Supplementary Figure S1).

### 3.2 Optimization of sample preparation

Two methods were used to optimize the treatment of plasma samples, namely, protein precipitation method and liquid-liquid extraction method. The optimal extraction recovery and matrix effect of each analyte were 85.0%–115.0%. It was found that the extraction recovery of each analyte was very small when ethyl acetate was used for liquid-liquid extraction, while the extraction recovery was higher when methanol, acetonitrile and methanol-acetonitrile mixed solution was used as the extraction solvent. Further optimization showed that the average recovery rate of methanol/acetonitrile (1/1, *v/v*) was more than 85%, which was suitable for the determination of biological samples. On this basis, vortex time (1 min, 3 min, 5 min) and resolution solvent (methanol, 70% methanol, 50% methanol) were optimized. The results showed that the extraction recovery and matrix effect were the best when 70% methanol was resolution and vortex time was 5 min. In addition, it has been reported that baicalin and wogonoside are not stable under alkaline and light and heat conditions (Wang et al., 2008; Feng et al., 2017). When a certain amount of formic acid was added into methanol-acetonitrile protein precipitation method, the extraction recovery of each analyte was improved obviously. Altogether, 10% (*v*/*v*) formic acid, methanol/acetonitrile (1/1, *v*/*v*), vortex time (5 min) and reconstitution solvent (70% methanol) were selected as the best processing conditions for plasma samples (Supplementary Figure S2). The extraction recovery rate and matrix effect of each analyte were in line with the determination requirements of biological samples.

### 3.3 Method validation

#### 3.3.1 Specificity

**FIGURE 1:**
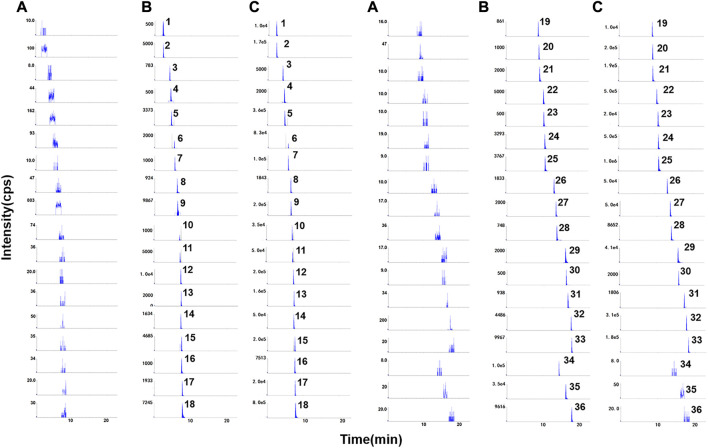
Representative chromatograms of **(A)** blank rat plasma, **(B)** blank rat plasma spiked with mixed standard compounds at LLOQs and ISs, **(C)** real samples after administration of QJYQ. 1–36 were harpagide, protocatechuic acid, atractyloside A, catechin, chlorogenic acid, cryptochlorogenic acid, verbenalin, paeoniflorin, *p*-Coumaric acid, ferulic acid, sinapic acid, vitexin, liquiritin, liquiritin apioside, isoferulic acid, hyperoside, cimifugin, scutellarin, quercitrin, hesperidin, apigenin-7-*O*-glucuronide, isoliquiritin apioside, ononin, baicalin, isoliquiritin, harpagoside, wogonoside, naringenin, baicalein, ginsenoside Rf, ginsenoside Rh1, glycyrrhizic acid, wogonin, icariin (IS), isopimpinellin (IS) and astragaloside II (IS), respectively.

According to the typical chromatograms of the blank sample, blank plasma spiked with mixed standards and ISs and plasma sample, it can be seen that the analytes were well separated without interference from endogenous substances or metabolites (Figure 1).

#### 3.3.2 Linearity and LLOQ

The curve of 33 analytes was fitted using a weighted (1/X or 1/X^2^) least squares linear regression approach, and a satisfactory correlation coefficient (r > 0.9928) was achieved. The LLOQs for 33 components ranged from 0.4–60.5 ng/mL (Supplementary Table S2).

#### 3.3.3 Precision and accuracy

The accuracy varied from 81.4% to 115.0%, and the RSDs for both intra-day and inter-day was under 15.0%. The outcomes showed that the developed method was reliable for determination of the 33 compounds in rat plasma (Table 1).

**TABLE 1:** Extraction recovery, matrix effect and precision of 33 analytes from QJYQ and 3 ISs.

No	Compounds	Concentration (ng/mL)	Intra-day		Inter-day		Recovery		Matrix effect	
			Accuracy (%)	RSD (%)	Accuracy (%)	RSD (%)	Accuracy (%)	RSD (%)	Normalized matrix factor	RSD (%)
1	harpagide	1.56	115.0	5.1	94.7	11.2	85.0	14.2	1.03	13.4
		12.5	91.3	13.6	88.2	13.7	87.9	3.8	1.02	11.3
		100	95.6	4.6	103.0	4.4	94.8	5.5	0.93	5.1
		500	85.2	7.6	91.0	7.0	88.4	7.8	0.94	9.2
2	protocatechuic acid	1.56	102.0	13.1	90.9	10.7	87.5	7.9	0.95	14.7
		12.5	97.1	7.8	90.0	10.5	85.4	13.6	0.95	14.7
		100	85.0	5.1	95.5	11.9	83.9	3.7	1.12	5.0
		500	92.7	5.8	90.2	7.2	97.7	8.2	0.93	6.8
3	atractyloside A	50	83.2	5.1	94.7	4.4	72.9	7.3	1.12	9.7
		100	94.8	7.5	96.1	5.7	75.2	4.1	1.03	7.9
		200	93.0	3.3	92.0	6.6	87.9	6.2	1.03	11.7
		500	102.0	12.2	101.0	10.7	92.7	14.7	0.97	7.4
4	catechin	12.5	96.5	4.4	94.3	7.0	95.6	4.0	1.10	7.0
		50	95.1	3.2	104.0	10.0	83.4	8.6	0.97	4.3
		100	100.0	15.0	92.2	11.0	85.9	6.8	1.06	3.7
		500	89.6	11.2	86.0	5.1	97.3	2.6	0.96	4.5
5	chlorogenic acid	12.5	91.2	9.5	94.5	11.8	73.4	5.2	1.12	4.8
		50	90.9	9.9	86.3	7.5	74.5	4.2	1.00	5.3
		100	102.0	11.5	111.0	4.5	96.2	5.6	1.00	9.0
		500	108.0	10.6	93.2	6.0	94.4	6.0	0.90	9.2
6	cryptochlorogenic acid	12.5	91.3	8.7	109.0	8.2	106.0	8.0	1.13	8.2
		50	113.0	8.0	110.0	10.7	73.6	4.5	1.03	3.2
		100	101.0	9.7	101.0	1.9	73.8	5.5	1.12	11.3
		500	111.0	4.0	97.1	5.5	103.0	6.6	0.89	5.3
7	verbenalin	12.5	106.0	14.2	100.0	11.2	86.4	7.2	0.95	6.5
		50	90.5	5.6	88.6	7.0	80.4	8.1	1.15	10.5
		100	90.6	4.9	91.5	6.1	79.0	5.9	1.11	10.4
		500	100.0	9.3	89.5	6.9	80.6	5.6	0.93	5.4
8	paeoniflorin	12.5	102.0	7.4	101.0	10.0	96.6	7.5	0.93	6.7
		50	92.5	5.5	93.3	7.4	77.9	13.4	1.10	2.9
		100	110.0	5.7	110.0	5.4	81.9	10.6	1.00	5.5
		500	91.2	10.6	90.6	12.0	99.6	5.3	1.13	6.5
9	*p*-Coumaric acid	1.56	111.0	15.0	108.0	14.0	93.9	8.8	1.01	13.9
		12.5	109.0	5.9	106.0	12.1	76.0	8.8	1.11	11.2
		100	93.6	4.0	98.8	5.1	88.7	2.9	1.12	3.4
		500	104.0	5.0	92.3	9.1	95.2	3.1	1.07	5.0
10	ferulic acid	12.5	95.7	9.7	101.0	14.7	79.8	15.0	1.12	6.6
		50	91.2	5.9	90.1	6.1	81.4	14.7	1.13	4.1
		100	101.0	8.1	86.8	12.4	82.6	5.8	1.02	1.9
		500	95.4	5.7	95.6	8.6	78.3	7.2	1.03	4.5
11	sinapic acid	1.56	93.3	10.8	102.0	13.2	84.3	3.6	1.00	9.0
		12.5	106.0	12.9	89.2	11.6	83.9	3.6	0.93	11.4
		100	94.2	8.8	90.0	6.3	74.8	9.6	1.10	4.9
		500	89.5	7.8	94.4	8.4	76.2	5.2	1.03	3.2
12	vitexin	1.56	106.0	5.1	98.1	13.8	101.0	6.2	1.13	7.1
		12.5	112.0	13.9	93.2	10.2	82.6	9.3	1.13	7.6
		100	92.5	6.6	95.1	6.5	85.1	8.4	1.01	12.3
		500	96.6	4.0	108.0	7.0	98.3	3.4	1.01	4.4
13	liquiritin	1.56	106.0	9.0	92.4	4.7	86.1	8.1	1.01	8.3
		12.5	105.0	7.5	89.5	8.8	80.5	12.2	1.01	8.5
		100	91.8	7.0	90.0	4.8	73.0	3.0	1.12	5.0
		500	95.8	8.4	93.4	10.0	74.0	2.9	1.03	3.1
14	liquiritin apioside	12.5	91.6	15.0	86.0	7.3	81.1	9.1	0.94	7.6
		50	102.0	5.7	90.3	11.2	76.4	13.9	0.95	12.8
		100	95.4	7.6	102.0	10.7	104.0	10.9	0.88	12.3
		500	99.1	4.0	93.0	6.0	103.0	8.6	0.97	4.7
15	isoferulic acid	50	98.5	13.5	94.2	8.0	90.3	5.4	0.98	7.0
		100	89.5	7.2	93.2	10.4	87.9	3.0	0.88	14.0
		200	105.0	9.1	94.6	9.9	89.2	10.1	0.96	9.3
		500	95.6	6.8	103.0	10.5	82.9	14.9	1.00	11.0
16	hyperoside	1.56	88.9	6.2	97.5	12.8	83.4	6.2	1.02	6.5
		12.5	110.0	11.2	103.0	5.0	85.7	3.4	1.15	4.5
		100	96.2	3.1	109.0	7.0	99.9	4.1	0.93	6.3
		500	108.0	4.4	91.1	9.5	100.0	3.6	1.11	7.1
17	cimifugin	1.56	108.0	8.8	81.4	2.5	90.3	4.2	1.13	4.4
		12.5	90.1	7.3	101.0	5.3	88.8	5.4	1.10	5.8
		100	102.0	7.4	100.0	11.0	90.9	3.5	1.10	5.1
		500	85.0	11.6	100.0	12.2	91.8	3.2	1.00	4.0
18	scutellarin	50	86.9	5.6	91.5	4.2	85.4	13.5	1.08	7.1
		100	95.6	9.7	93.6	8.1	81.2	14.0	1.11	9.2
		200	91.4	8.1	103.0	8.4	89.6	11.4	0.89	4.3
		500	114.0	5.0	112.0	6.2	105.0	1.4	0.98	12.3
19	quercitrin	1.56	107.0	11.5	95.5	2.9	112.0	6.2	1.08	8.2
		12.5	88.9	15.0	89.1	6.0	84.0	6.3	1.01	4.0
		100	105.0	7.8	106.0	9.9	86.6	2.6	1.01	11.2
		500	95.2	6.7	91.2	10.2	110.0	6.1	0.93	13.7
20	hesperidin	12.5	102.0	12.6	94.4	13.4	86.8	6.5	0.90	10.3
		50	92.7	10.6	96.7	9.3	94.9	5.8	1.10	1.9
		100	95.3	6.5	104.0	10.4	113.0	4.3	1.10	1.9
		500	95.9	8.2	85.0	8.9	79.1	5.2	0.95	5.9
21	apigenin-7-*O*-glucuronide	1.56	104.0	14.1	84.2	4.1	85.1	10.3	0.95	11.5
		12.5	94.0	5.2	94.8	7.8	78.2	8.3	1.02	12.7
		100	86.7	5.4	93.0	7.3	92.9	2.3	1.10	5.7
		500	106.0	7.1	101.0	12.4	84.5	1.9	0.92	7.6
22	isoliquiritin apioside	1.56	86.9	4.6	95.2	7.9	98.1	6.4	1.12	6.5
		12.5	89.9	7.1	112.0	6.0	80.7	4.8	1.02	4.4
		100	101.0	8.1	107.0	2.0	100.0	5.6	1.03	2.7
		500	95.2	2.0	99.2	15.0	82.9	1.8	1.00	11.4
23	ononin	1.56	100.0	10.3	90.4	6.4	96.5	8.5	1.10	7.3
		12.5	101.0	11.8	97.8	5.9	77.3	6.8	1.14	4.6
		100	95.2	13.7	90.7	5.7	79.3	4.9	1.04	11.4
		500	102.0	5.0	94.8	1.9	98.4	2.6	1.04	3.3
24	baicalin	15.6	114.0	12.5	112.0	13.0	85.5	1.9	1.10	13.4
		125	94.2	3.1	103.0	4.8	94.8	4.0	1.07	14.4
		1000	98.2	6.3	93.2	6.0	85.1	4.2	1.13	6.7
		5000	112.0	7.4	97.9	8.2	96.9	3.2	0.93	6.7
25	isoliquiritin	1.56	88.1	10.6	100.0	6.4	98.7	4.6	1.10	6.8
		12.5	90.8	5.2	94.4	3.4	82.5	7.3	1.10	3.5
		100	96.7	9.3	104.0	13.0	85.0	5.1	1.13	1.4
		500	98.6	10.0	111.0	8.2	100.0	3.4	0.90	6.4
26	harpagoside	1.56	107.0	7.9	84.6	4.2	76.1	9.8	1.10	10.9
		12.5	106.0	5.9	99.0	5.1	75.6	9.2	1.03	6.2
		100	94.1	4.3	90.8	7.8	98.3	5.6	1.11	4.8
		500	88.8	3.7	91.2	10.9	101.0	7.4	0.93	4.8
27	wogonoside	125	94.9	9.9	111.0	10.7	74.8	2.0	0.91	13.0
		500	112.0	6.0	108.0	1.0	80.7	14.3	1.00	13.0
		1000	87.3	14.4	93.3	5.5	101.0	9.6	1.10	8.2
		5000	101.0	6.4	91.6	5.6	83.3	7.1	0.93	9.3
28	naringenin	12.5	100.0	5.6	98.6	8.2	100.0	6.0	1.15	4.9
		50	85.5	4.2	89.1	5.3	84.6	5.8	1.14	4.8
		100	88.6	5.6	101.0	4.4	86.6	4.0	0.94	10.6
		500	90.0	7.0	93.5	8.1	102.0	5.4	1.02	3.6
29	baicalein	12.5	104.0	7.4	107.0	6.5	104.0	10.3	1.13	6.5
		50	93.3	4.0	105.0	7.5	98.4	8.0	0.85	14.8
		100	92.3	3.7	112.0	8.3	101.0	4.6	0.96	4.6
		500	91.0	5.2	96.1	5.4	104.0	10.3	0.96	8.4
30	ginsenoside Rf	12.5	94.2	9.7	96.5	8.8	79.9	14.2	0.98	14.5
		50	106.0	8.6	102.0	6.0	113.0	14.9	0.88	8.4
		100	95.0	7.3	107.0	7.4	85.0	11.6	1.03	10.7
		500	105.0	8.4	97.6	12.6	106.0	13.9	1.12	8.2
31	ginsenoside Rh1	50	102.0	7.3	94.6	14.6	113.0	14.3	1.00	12.6
		100	114.0	3.0	88.5	13.1	90.9	11.7	0.90	8.2
		200	89.6	6.8	86.9	5.1	95.7	8.7	1.12	6.5
		500	88.7	10.5	85.7	9.6	114.0	7.3	0.87	5.4
32	glycyrrhizic acid	12.5	91.6	15.0	86.0	7.3	81.1	9.1	0.98	7.6
		50	102.0	5.7	90.3	11.2	76.4	13.9	0.99	12.8
		100	95.4	7.5	102.0	10.7	104.0	10.9	0.89	12.3
		500	99.1	4.0	93.0	6.0	103.0	8.6	0.92	4.7
33	wogonin	1.56	82.3	3.9	100.0	13.0	110.0	15.0	0.93	6.8
		12.5	105.0	9.9	95.6	14.3	99.8	9.5	1.03	11.1
		100	90.6	5.4	87.5	6.0	84.0	1.4	1.13	1.9
		500	95.0	9.1	100.0	4.0	83.9	2.5	1.05	10.1
34	icariin (IS)	100	-	-	-	-	94.8	6.2	0.94	6.2
35	isopimpinellin (IS)	100	-	-	-	-	93.8	12.2	1.03	11.1
36	astragaloside II (IS)	100	-	-	-	-	88.8	9.9	1.02	9.9

#### 3.3.4 Recovery and matrix effect

The extraction recoveries and matrix effect of all analytes ranged from 72.9% to 115.0% in four levels of QC samples. The RSDs were less than 15.0%. The results show that this plasma sample pre-treatment method can meet the needs of multi-component determination *in vivo* and pharmacokinetic studies (Table 1).

#### 3.3.5 Stability

The 33 compounds were stable in rat plasma under the following conditions (room temperature for 24 h, auto-sampler for 24 h, three freeze-thaw cycles and stored at −80°C for 1 month). The stability of the working solution was relatively stable for all the target analytes. The results indicated that the method could be used to simultaneous determine the 33 compounds in rat plasma (Table 2).

**TABLE 2:** Stability of 33 analytes in QJYQ.

No	Compounds	Concentration (ng/mL)	Autosampler for 24 h		Room temperature for 24 h		Freeze thaw cycles		−80°C for 1month		Working solution	
			Accuracy (%)	RSD (%)	Accuracy (%)	RSD (%)	Accuracy (%)	RSD (%)	Accuracy (%)	RSD (%)	Accuracy (%)	RSD (%)
1	harpagide	1.56	93.7	4.3	90.2	9.5	97.3	12.5	105.0	10.7	82.1	13.0
		12.5	88.1	12.3	92.7	14.5	102.0	14.0	92.9	6.2	107.0	12.2
		100	96.1	15.0	95.6	4.6	96.3	11.4	88.3	12.3	92.4	12.2
		500	93.4	10.8	96.4	13.9	105.0	6.4	92.7	12.4	101.0	10.8
2	protocatechuic acid	1.56	103.0	15.0	110.0	12.6	103.0	14.0	80.4	10.2	82.3	8.2
		12.5	86.0	11.4	91.0	13.2	101.0	14.3	114.0	13.2	104.0	9.9
		100	98.5	2.2	106.0	7.2	90.0	9.4	100.0	6.7	92.6	3.5
		500	85.2	4.1	85.3	12.2	89.0	10.8	91.2	8.7	88.9	13.0
3	atractyloside A	50	107.0	5.4	97.6	3.1	84.6	2.9	89.7	10.6	88.5	11.2
		100	94.4	9.0	104.0	8.3	101.0	11.4	96.2	11.5	104.0	13.8
		200	91.5	9.3	89.9	7.9	97.0	3.3	93.4	8.4	87.0	6.4
		500	103.0	5.8	91.2	3.9	102.0	10.8	92.2	5.0	97.2	6.9
4	catechin	12.5	92.6	10.6	84.7	8.6	96.6	11.6	94.6	7.7	91.6	15.0
		50	108.0	5.6	92.6	8.2	96.1	3.8	106.0	9.6	106.0	5.9
		100	97.4	4.4	98.2	8.5	95.2	10.6	102.0	14.2	97.4	9.6
		500	96.1	4.2	104.0	6.1	90.1	6.0	95.1	12.5	100.0	4.7
5	chlorogenic acid	12.5	94.6	10.2	97.3	9.4	100.0	10.3	98.3	10.3	108.0	5.0
		50	97.5	9.5	106.0	8.0	105.0	11.2	115.0	8.0	107.0	3.5
		100	96.9	8.0	105.0	3.8	95.5	5.5	102.0	3.2	86.8	2.0
		500	85.5	10.7	94.3	6.7	96.9	6.0	94.3	13.7	85.9	10.8
6	cryptochlorogenic acid	12.5	94.6	7.9	90.6	14.0	84.5	10.7	90.6	6.9	87.6	9.9
		50	93.1	6.3	91.2	12.7	86.6	4.9	107.0	12.9	95.0	7.8
		100	92.7	8.1	102.0	10.5	88.9	13.0	110.0	5.4	94.6	8.3
		500	109.0	14.0	100.0	11.2	98.7	7.6	104.0	8.6	96.8	5.4
7	verbenalin	12.5	84.9	4.4	104.0	13.1	93.8	13.5	89.1	10.3	83.3	14.4
		50	92.5	6.8	88.9	4.3	105.0	10.7	105.0	12.2	89.5	8.3
		100	89.7	7.3	91.2	4.6	87.8	6.51	95.7	12.2	86.2	6.0
		500	88.3	6.1	104.0	12.3	88.5	12.3	104.0	15.0	96.4	1.9
8	paeoniflorin	12.5	86.8	5.6	106.0	9.0	97.6	11.6	97.6	12.6	106.0	8.7
		50	97.6	8.3	106.0	5.5	94.1	12.8	96.4	13.8	97.6	12.6
		100	111.0	6.0	86.8	4.0	93.4	14.2	95.2	10.2	98.1	13.8
		500	92.0	5.0	92.2	11.2	93.1	4.5	95.3	6.5	93.2	10.2
9	*p*-Coumaric acid	1.56	83.2	6.8	82.7	12.2	95.1	14.0	110.0	5.8	81.9	9.5
		12.5	102.0	12.8	96.1	14.8	95.2	14.0	85.1	11.8	107.0	11.2
		100	96.9	9.6	98.7	6.4	87.0	10.5	115.0	11.8	95.9	3.1
		500	115.0	5.3	97.8	4.0	88.7	10.8	109.0	10.2	92.8	5.2
10	ferulic acid	12.5	84.5	5.5	104.0	13.7	96.0	14.4	115.0	14.3	87.7	12.6
		50	86.9	6.0	96.8	6.5	98.3	10.3	90.9	15.0	96.8	6.5
		100	91.5	10.7	86.7	3.1	115.0	8.0	95.9	7.6	88.3	4.5
		500	93.6	4.9	89.2	8.3	102.0	3.2	107.0	14.7	96.7	9.2
11	sinapic acid	1.56	95.8	12.9	113.0	5.4	106.0	13.1	105.0	14.2	100.0	10.4
		12.5	95.9	3.6	90.8	9.1	97.9	13.9	93.3	11.4	90.2	14.0
		100	90.5	3.4	85.2	7.4	113.0	11.3	112.0	9.4	94.2	8.8
		500	94.0	5.6	89.6	5.0	101.0	5.3	103.0	10.1	87.0	8.7
12	vitexin	1.56	95.0	10.4	92.0	7.5	103.0	7.3	90.4	14.2	95.6	6.9
		12.5	93.5	12.3	105.0	4.2	92.0	4.4	106.0	4.7	88.3	6.5
		100	93.2	10.0	88.3	11.2	85.5	11.4	93.2	10.1	89.9	9.0
		500	92.4	9.3	107.0	9.1	108.0	7.8	92.6	2.5	106.0	8.8
13	liquiritin	1.56	84.0	13.3	111.0	14.9	110.0	12.9	104.0	13.5	99.4	14.9
		12.5	108.0	9.6	94.4	12.9	104.0	14.5	94.1	6.9	94.1	6.8
		100	94.1	2.2	90.6	9.2	85.8	12.1	103.0	9.2	100.0	7.2
		500	91.2	7.0	89.3	10.6	87.0	9.0	108.0	14.3	86.6	15.0
14	liquiritin apioside	1.56	84.7	14.9	102.0	7.0	90.2	8.1	100.0	10.7	100.0	4.8
		12.5	98.8	4.2	86.6	15.0	100.0	11.7	102.0	11.5	115.0	3.7
		100	87.2	9.3	89.2	5.6	104.0	12.3	99.1	12.9	87.2	9.3
		500	97.9	7.0	95.0	6.4	101.0	10.5	105.0	7.9	87.9	5.6
15	isoferulic acid	50	97.1	7.8	108.0	6.9	95.7	13.7	90.8	7.4	100.0	8.3
		100	88.3	11.0	87.5	6.2	86.2	3.8	85.5	5.4	95.7	14.9
		200	102.0	10.3	87.2	10.0	97.1	6.0	105.0	12.6	104.0	12.7
		500	104.0	11.2	97.1	13.5	95.6	14.2	106.0	11.1	101.0	11.5
16	hyperoside	1.56	111.0	6.1	91.6	15.0	97.6	12.6	106.0	9.0	97.6	12.6
		12.5	101.0	3.3	102.0	5.6	98.4	14.8	100.0	7.5	92.1	9.8
		100	90.5	6.2	95.4	7.6	93.5	13.2	88.8	7.0	91.2	10.2
		500	95.8	7.4	99.1	4.0	95.3	2.5	102.0	7.6	95.0	8.5
17	cimifugin	1.56	102.0	7.5	92.4	8.0	84.5	14.2	83.9	9.5	90.5	3.0
		12.5	91.1	6.5	95.6	7.4	92.0	7.02	96.3	9.2	100.0	6.5
		100	100.0	8.2	98.0	2.0	95.7	8.98	99.6	10.5	101.0	9.6
		500	102.0	11.2	98.6	10.4	93.2	14.2	95.2	7.2	86.7	10.4
18	scutellarin	50	102.0	5.1	91.2	3.9	102.0	10.8	92.2	5.0	97.2	6.8
		100	109.0	6.2	101.0	8.1	111.0	9.1	101.0	3.2	108.0	4.1
		200	94.7	5.7	90.6	6.5	85.4	8.7	90.4	2.8	99.4	5.7
		500	101.0	14.6	105.0	9.0	112.0	12.7	103.0	11.6	100.0	12.6
19	quercitrin	1.56	95.2	4.3	85.2	3.1	86.2	3.8	86.7	5.6	92.6	12.0
		12.5	94.7	9.5	87.1	2.0	90.0	4.6	97.1	6.0	101.0	6.6
		100	98.3	9.0	99.6	11.2	100.0	6.2	95.6	14.2	90.4	8.6
		500	108.0	10.3	98.0	11.4	89.3	11.0	96.0	14.4	99.1	4.1
20	hesperidin	12.5	82.1	12.0	84.1	4.2	94.3	13.7	81.8	12.9	81.7	10.7
		50	100.0	9.0	93.7	11.3	85.4	13.3	85.8	8.7	109.0	9.2
		100	96.0	13.7	92.3	13.3	95.7	14.6	93.1	14.9	102.0	10.1
		500	87.8	7.5	92.3	7.2	89.1	12.9	93.6	8.8	91.0	10.1
21	apigenin-7-*O*-glucuronide	1.56	103.0	3.7	114.0	9.2	109.0	12.1	104.0	15.0	83.6	3.8
		12.5	93.2	10.2	92.2	6.4	85.6	13.2	86.0	11.5	112.0	9.6
		100	94.1	5.7	90.4	9.5	86.4	11.1	85.0	12.3	88.5	4.4
		500	85.5	4.1	104.0	11.9	88.3	8.5	108.0	6.0	93.2	5.9
22	isoliquiritin apioside	1.56	96.2	9.8	84.3	10.0	97.1	4.8	91.6	15.0	96.2	9.8
		12.5	87.1	6.8	103.0	9.0	88.6	13.7	102.0	6.7	98.1	6.8
		100	85.6	14.7	102.0	8.3	107.0	12.4	95.4	5.6	97.6	7.2
		500	106.0	6.4	99.5	7.5	96.3	11.3	99.1	14.0	100.0	6.1
23	ononin	1.56	106.0	4.4	98.6	6.1	95.6	8.9	111.0	9.6	84.3	10.7
		12.5	95.3	13.9	109.0	8.3	108.0	15.0	92.2	6.6	87.5	8.0
		100	94.4	6.1	86.9	2.9	99.0	2.4	95.4	3.4	104.0	9.4
		500	104.0	6.1	93.2	5.9	106.0	11.8	98.2	7.0	91.3	6.9
24	baicalin	15.6	111.0	12.3	98.5	5.7	101.0	15.0	98.9	11.2	83.1	10.3
		125	113.0	15.0	104.0	6.7	89.3	6.9	98.1	12.9	105.0	8.6
		1000	94.2	7.3	96.8	6.1	89.7	8.4	91.4	11.3	93.9	6.9
		5000	86.8	9.3	89.0	12.9	111.0	11.7	90.3	8.2	87.7	7.9
25	isoliquiritin	1.56	99.3	5.3	88.9	6.1	95.5	6.5	83.8	8.0	106.0	5.7
		12.5	108.0	4.0	87.5	5.7	90.9	6.0	105.0	8.5	110.0	8.0
		100	107.0	3.2	96.6	8.9	94.5	10.7	106.0	7.0	98.1	3.1
		500	96.3	2.7	96.7	5.3	91.6	4.9	95.0	6.2	93.3	10.7
26	harpagoside	1.56	105.0	8.1	95.0	7.3	91.5	10.6	86.6	15.0	83.8	7.0
		12.5	101.0	7.0	108.0	8.6	98.1	13.8	102.0	5.7	105.0	7.5
		100	93.8	6.3	100.0	5.9	93.2	10.2	95.4	7.6	101.0	6.0
		500	102.0	12.5	95.7	8.6	95.1	6.5	99.1	4.0	88.5	6.8
27	wogonoside	62.5	89.7	9.7	83.2	12.7	84.2	11.4	83.7	14.4	91.0	3.3
		500	105.0	7.2	93.4	8.0	97.1	12.1	113.0	8.2	113.0	4.5
		1000	98.7	7.2	85.6	11.6	95.1	7.0	98.0	8.3	105.0	9.4
		5000	97.5	10.6	111.0	11.7	90.9	5.9	89.1	2.8	108.0	8.9
28	naringenin	12.5	101.0	9.3	94.4	12.2	97.6	12.5	85.7	5.7	93.5	7.3
		50	98.3	7.9	101.0	5.3	104.0	13.4	98.2	5.6	102.0	14.8
		100	99.5	6.6	92.9	7.8	101.0	6.4	97.4	8.4	85.6	11.4
		500	107.0	5.7	95.2	9.8	102.0	9.1	94.2	8.1	98.2	2.9
29	baicalein	12.5	97.0	12.0	91.4	12.6	97.4	6.6	99.7	10.5	92.0	12.6
		50	92.3	7.5	85.5	6.0	96.3	5.5	90.6	9.5	91.3	11.5
		100	92.4	8.6	95.6	6.2	98.2	8.4	95.5	7.2	92.9	8.2
		500	93.2	2.9	91.0	9.2	94.0	3.9	104.0	8.6	89.7	6.1
30	ginsenoside Rf	12.5	112.0	6.8	96.1	13.8	97.2	13.0	97.1	15.0	91.9	2.9
		50	98.9	4.6	92.7	6.2	88.0	2.6	89.6	8.8	94.9	5.1
		100	88.2	3.3	96.8	5.7	89.7	5.9	105.0	7.2	110.0	2.2
		500	106.0	12.7	113.0	8.6	106.0	8.1	112.0	7.2	102.0	3.2
31	ginsenoside Rh1	50	86.5	15.0	100.0	10.5	101.0	6.5	107.0	9.2	106.0	8.4
		100	90.8	14.2	96.5	8.2	100.0	8.7	93.5	3.9	95.1	6.5
		200	96.5	7.4	90.2	9.1	101.0	3.4	101.0	3.6	107.0	10.9
		500	108.0	6.6	98.7	6.3	94.1	15.0	114.0	12.5	107.0	7.4
32	glycyrrhizic acid	12.5	94.4	11.3	101.0	9.1	84.6	14.3	85.6	9.5	101.0	13.8
		50	100.0	1.4	94.6	3.1	102.0	2.9	89.7	10.6	88.5	11.2
		100	94.3	2.0	107.0	8.3	101.0	11.4	96.2	11.5	104.0	13.8
		500	94.5	6.1	89.9	7.9	97.0	3.4	93.4	8.4	87.0	6.4
33	wogonin	1.56	82.8	4.1	98.0	8.5	86.4	7.0	111.0	14.1	98.0	8.5
		12.5	111.0	7.8	94.1	10.0	89.6	10.7	85.1	8.8	98.9	13.2
		100	102.0	5.6	89.8	3.8	104.0	10.3	96.0	14.4	85.8	2.9
		500	90.5	6.0	106.0	13.5	91.8	3.3	87.4	8.1	100.0	4.0
34	icariin (IS)	100	114.0	4.9	102.0	7.5	86.9	8.2	107.0	2.6	94.8	14.0
35	isopimpinellin (IS)	100	98.6	7.3	96.5	7.2	88.2	7.3	109.0	8.6	93.5	9.6
36	astragaloside II(IS)	100	105.0	9.6	102.0	5.2	105.0	5.4	115.0	11.0	96.7	10.8

### 3.4 Pharmacokinetic analysis

**FIGURE 2:**
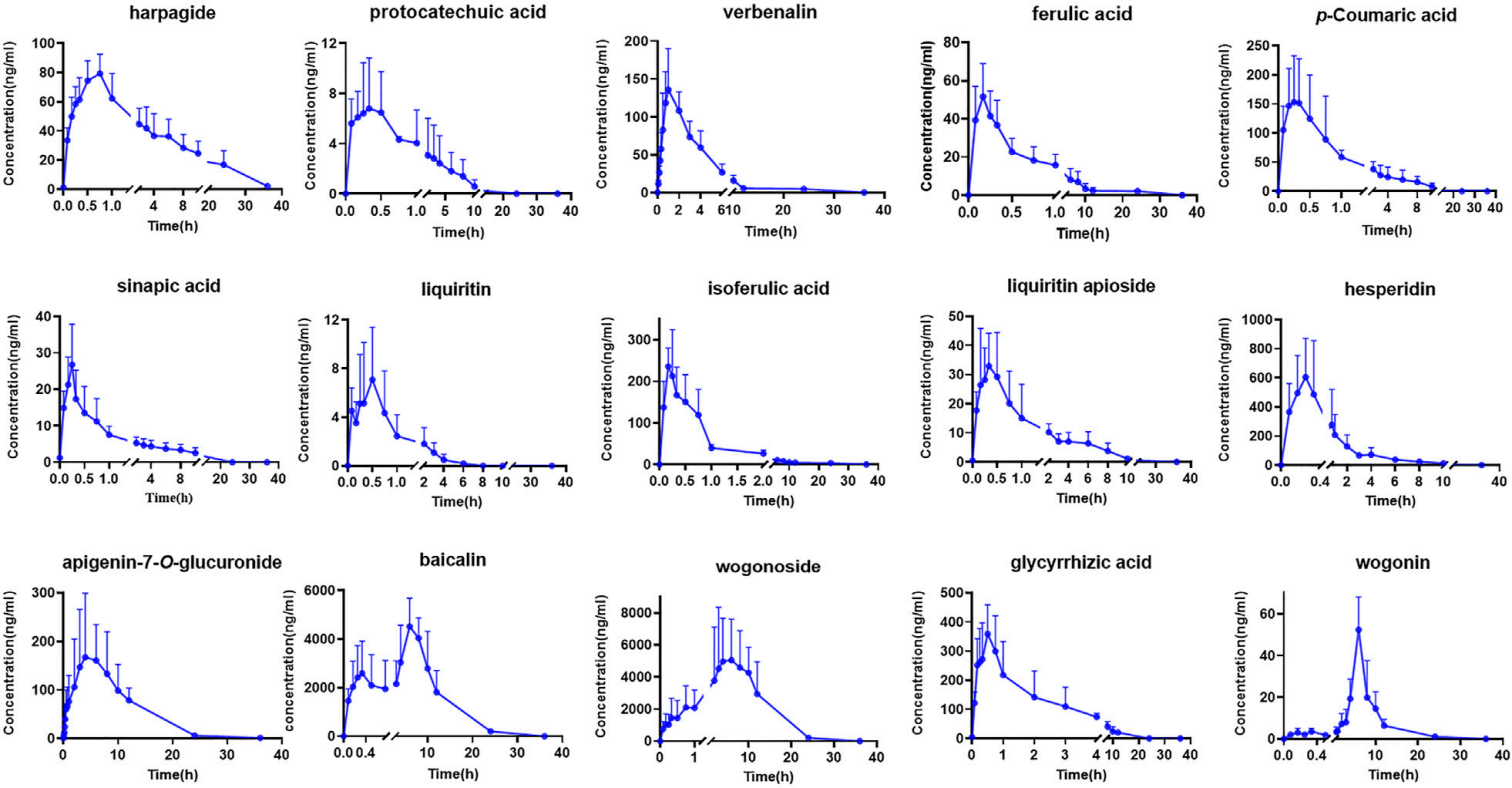
The mean plasma concentration-time of 15 compounds in rats after oral administration of QJYQ (n = 8, Mean ± SD).

Although TCMs composition was complex, only some components with certain concentration could play the corresponding curative effect when components were absorbed into the blood. Therefore, components that have absorbed into the blood with large plasma exposure could be used as indicators for evaluating their quality standards (He et al., 2018; Zhang et al., 2018). In this research, a total of 15 major constituents of QJYQ with large plasma exposure were obtained. The results showed the mean plasma concentration-time profiles and the major pharmacokinetic parameters of the 15 analytes (n = 8) (Figure 2; Table 3).

**TABLE 3:** Pharmacokinetic parameters of 15 components in plasma of QJYQ (n = 8, mean ± SD).

No	Compounds	Cmax (ng/mL)	Tmax (h)	T1/2 (h)	AUC_(0-t)_ (ng/mL·h)	AUC_(0-∞)_ (ng/mL·h)	MRT_(0-t)_ (h)	MRT _(0-∞)_ (h)
1	harpagide	79.38 ± 7.71	0.75 ± 0.16	7.62 ± 3.88	693.87 ± 217.37	723.75 ± 209.89	10.67 ± 1.23	12.55 ± 1.09
2	protocatechuic acid	6.79 ± 4.35	0.45 ± 0.13	2.33 ± 0.52	40.82 ± 10.92	40.92 ± 17.29	5.30 ± 1.02	5.32 ± 1.53
3	verbenalin	135.83 ± 52.28	1.37 ± 0.80	8.37 ± 2.80	618.13 ± 161.10	669.34 ± 164.70	4.85 ± 0.77	7.95 ± 4.85
4	*p*-Coumaric acid	153.32 ± 33.38	0.24 ± 0.08	1.79 ± 0.38	380.52 ± 101.04	385.37 ± 126.12	3.03 ± 1.01	3.63 ± 1.57
5	ferulic acid	51.71 ± 10.68	0.19 ± 0.09	2.12 ± 0.07	173.23 ± 5.66	176.72 ± 28.57	8.40 ± 0.20	8.43 ± 0.53
6	sinapic acid	26.77 ± 7.87	0.22 ± 0.04	2.20 ± 0.13	86.98 ± 6.62	86.99 ± 6.62	6.28 ± 0.91	6.28 ± 0.91
7	liquiritin	7.08 ± 2.25	0.52 ± 0.10	4.30 ± 3.09	16.28 ± 5.11	16.41 ± 5.06	6.40 ± 0.98	6.80 ± 1.19
8	liquiritin apioside	32.92 ± 4.54	0.33 ± 0.02	10.72 ± 7.42	135.24 ± 23.80	140.73 ± 27.38	6.15 ± 1.76	8.04 ± 4.40
9	isoferulic acid	235.38 ± 81.07	0.19 ± 0.04	4.68 ± 0.41	347.19 ± 119.74	354.10 ± 132.49	5.25 ± 2.87	5.85 ± 3.26
10	hesperidin	604.36 ± 145.25	0.28 ± 0.14	1.94 ± 0.03	1058.05 ± 267.32	1058.06 ± 267.32	4.52 ± 0.54	4.52 ± 0.54
11	apigenin-7-*O*-glucuronide	167.50 ± 108.49	4.65 ± 2.79	4.17 ± 1.51	14603.11 ± 7523.95	15028.42 ± 7667.50	8.39 ± 1.62	8.62 ± 1.65
12	baicalin	4522.70 ± 3135.64	7.22 ± 1.41	3.09 ± 0.87	49313.96 ± 24881.42	49666.13 ± 24896.34	8.45 ± 0.58	8.62 ± 0.53
13	wogonoside	5052.10 ± 3277.54	5.89 ± 3.26	2.67 ± 0.24	73080.32 ± 34091.07	73104.07 ± 34093.59	8.39 ± 0.83	8.40 ± 0.83
14	glycyrrhizic acid	359.38 ± 147.21	0.46 ± 0.10	1.67 ± 0.16	1107.32 ± 138.04	1116.50 ± 228.15	5.30 ± 0.28	5.38 ± 1.16
15	wogonin	38.13 ± 10.55	6.29 ± 0.76	1.91 ± 0.09	288.10 ± 66.09	288.10 ± 66.09	8.27 ± 1.09	8.27 ± 1.09

The T_max_ of isoferulic acid, ferulic acid, sinapic acid, *p*-Coumaric acid, glycyrrhizic acid, liquiritin apioside, protocatechuic acid and hesperidin is less than 0.5 h, suggesting that these compounds could be rapidly absorbed into blood circulation system. The T_max_ of baicalin is 7.22 h ± 1.41, which was the longest.

Baicalin, wogonoside and hesperidin have large maximum plasma concentrations, which are 4522.70 ± 3135.64, 5052.10 ± 3277.54, 604.36 ± 145.25 ng/mL, respectively, and they are also the main components of QJYQ (Yang, et al., 2023). Baicalin, wogonoside and apigenin-7-*O*-glucuronide have greater plasma exposure. The MRT of harpagide, ferulic acid, apigenin-7-*O*-glucuronide, wogonin, wogonoside and baicalin are all greater than 8 h, suggesting that they take a long time to be eliminated *in vivo*.

Baicalin has a higher AUC_(0-∞)_, which is 49666.13 ± 24896.34 ng/L·h. It may be attributed to the high content of the compound and its interaction with other components (Yang, et al., 2023). The drug-time curves of baicalin showed double peaks, suggesting that baicalin can be reabsorbed *in vivo* through the enterohepatic circulation (Huang et al., 2019). This will undoubtedly increase the blood concentration of baicalin and maintain a high level, which will be beneficial to the therapeutic effect of QJYQ.

The double-peak or even triple-peak phenomenon of wogonoside may be due to the transformation of wogonoside and wogonin *in vivo* (Dai et al., 2015). The AUC_(0-∞)_ of wogonoside is 73104.07 ± 34093.59 ng/L·h, which is the largest among the tested ingredients. It has been found that the Caco-2 cell membrane permeability of wogonoside is greater than baicalin, indicating that the absorption of wogonoside is better than baicalin. AUC_(0-t)_ and AUC_(0-∞)_ confirmed the result (Cai et al., 2016). The MRT_(0-t)_ of wogonoside and wogonin are both larger, which may be related to the local circulation of intestinal cells.

It has been reported that chlorogenic acid may be metabolized to ferulic acid and isoferulic acid after entering the body, resulting in increased levels of ferulic acid and isoferulic acid *in vivo* (Wang, et al., 2018). In this study, chlorogenic acid was not detected, which may have been converted to ferulic acid and isoferulic acid. At the same time, ferulic acid and isoferulic acid had shorter peak time and higher content in this study, which may be attributed to the conversion of chlorogenic acid. Studies have shown that glycyrrhizic acid consists of one molecule of glycyrrhetinic acid and two molecules of glucuronic acid, which are easily lost *in vivo* by the hydrolysis of two molecules of glucuronic acid to form glycyrrhetinic acid (Du, et al., 2019). In this study, the peak time and residence time of glycyrrhizic acid are short, so the research on its metabolites *in vivo* needs further exploration.

In this experiment, we detected 33 compounds in rat plasma. Among them, 18 compounds were not detected, probably due to their low concentrations, low oral availability *in vivo,* or metabolization into other products, which need further study. In summary, a total of 15 major constituents of QJYQ with large plasma exposure were obtained, including baicalin, wogonoside, wogonin, apigenin-7-*O*-glucuronide, verbenalin, isoferulic acid, hesperidin, glycyrrhizic acid, liquiritin, sinapic acid, ferulic acid, *p*-Coumaric acid, protocatechuic acid, harpagide and liquiritin apioside. The pharmacokinetic results indicated that these compounds of QJYQ had high absorption concentration, large plasma exposure *in vivo*, which was beneficial for QJYQ to exert its efficacy.

## 4 Discussions

The pharmacokinetics of multiple components in plasma after oral administration of QJYQ in rats have not been investigated. In the present study, the pharmacokinetics of 33 components in plasma after oral administration of QJYQ were investigated. Compounds selection principles are as follows: firstly, based on the previous *in vitro* quantitative study of QJYQ, we found 50 quantifiable components, among which 33 components with high content were selected as indicators (Yang, et al., 2023). Secondly, it has been reported that these compounds can be absorbed into the blood and have high plasma exposure. For example, studies have shown that baicalin, baicalein, wogonoside and wogonin can be well absorbed into blood in the pharmacokinetics study of *Scutellariae radix* (He, et al., 2018). Some researchers have studied the pharmacokinetics of *verbenae herba*, showing that verbenalin has a large plasma exposure (Liu, et al., 2019). Finally, those compounds have potential activity, which is helpful to the further pharmacological studies of QJYQ (Yang et al., 2017; Yuan et al., 2018). Based on the above principles, we selected these 33 compounds to explore their processes *in vivo*.

Most of the reported methods for pharmacokinetic determination of TCMs compound preparations are HPLC or LC-MS/MS (Si, et al., 2008; Wang, et al., 2019). In this study, the established UHPLC-sMRM method has the advantages of low injection volume, short running time and wider linear range compared with the published method (Xing, et al., 2013; Guan, et al., 2017; Zhao, et al., 2019). Compared with the MRM scan mode, sMRM mode can analyze targeted ion pairs in a specific time window, improving the sensitivity of detection (Zhang, et al., 2015; Chen, et al., 2019). All in all, the established UHPLC-sMRM method is more rapid, simple, high selective and sensitive, which is conducive to the accurate quantitative analysis of multiple targets with different concentrations in the complex matrix of TCMs (Si, et al., 2008; Cai, et al., 2016; Wang, et al., 2019; Wu, et al., 2019) (Supplementary Table S3).

## 5 Conclusion

A rapid and sensitive UHPLC-sMRM was successfully established and validated for 33 components in rat plasma of QJYQ, showing its excellent precision, stability and recovery. Fifteen major constituents including wogonin, baicalin, sinapic acid, ferulic acid, hesperidin, wogonoside, apigenin-7-*O*-glucuronide, verbenalin, isoferulic acid, liquiritin, glycyrrhizic acid, liquiritin apioside, harpagide, *p*-Coumaric acid and protocatechuic acid of QJYQ with large plasma exposure were obtained. This experiment preliminarily provides reference for elucidating the pharmacodynamic substance basis, further study of human pharmacokinetics and design of rational drug regimen.

## Data Availability

The original contributions presented in the study are included in the article/Supplementary Material, further inquiries can be directed to the corresponding authors.
