# Supplementary figures and images for: Simultaneous determination of multiple components in rat plasma by UHPLC-sMRM for pharmacokinetic studies after oral administration of Qingjin Yiqi Granules
Source: Front Pharmacol. 2023 Apr 13;14:1155973. doi: 10.3389/fphar.2023.1155973 (PMC10133546; doi:10.3389/fphar.2023.1155973)

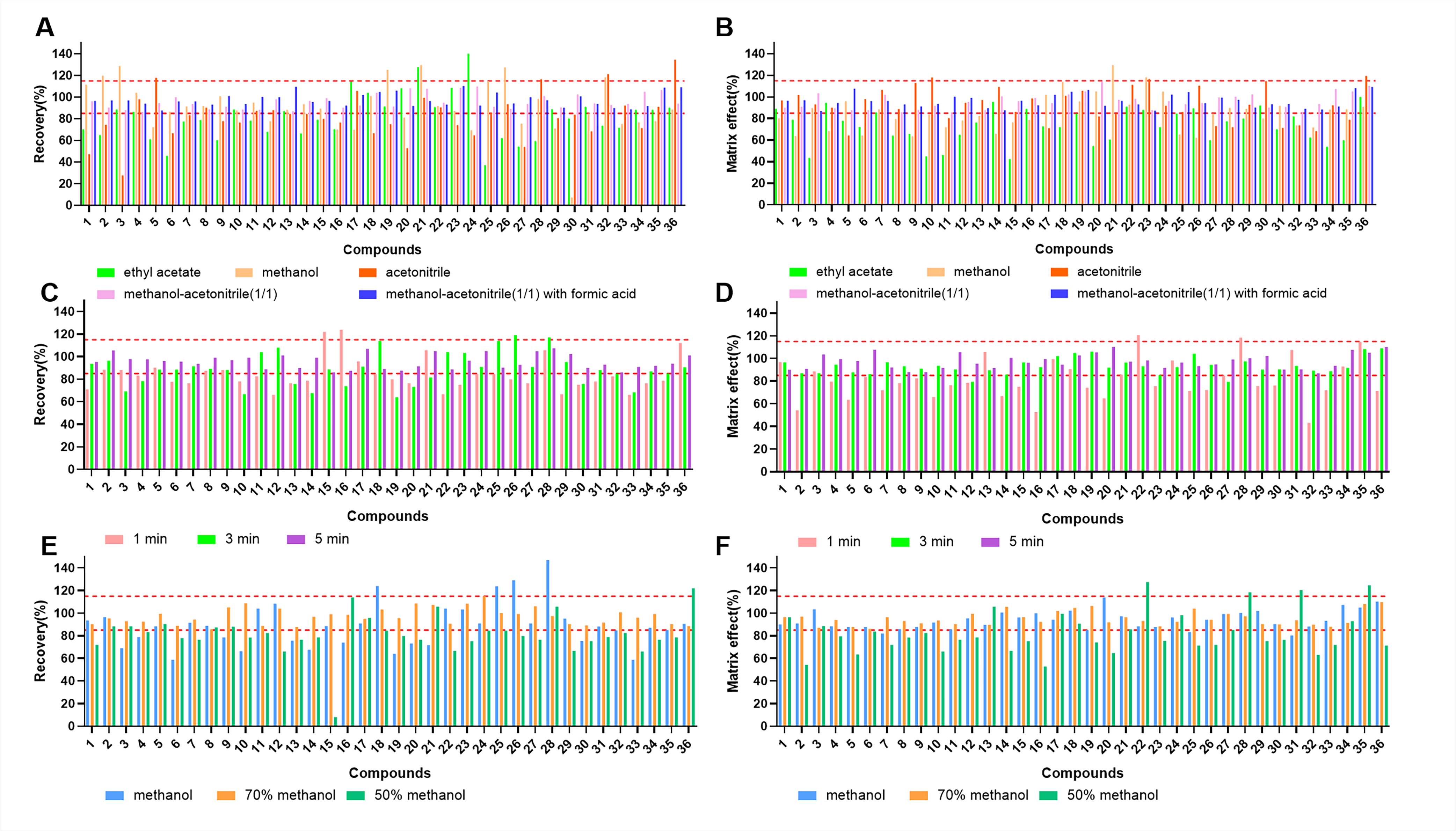

Supplement: Supplementary file 1 [file Image2.TIF]

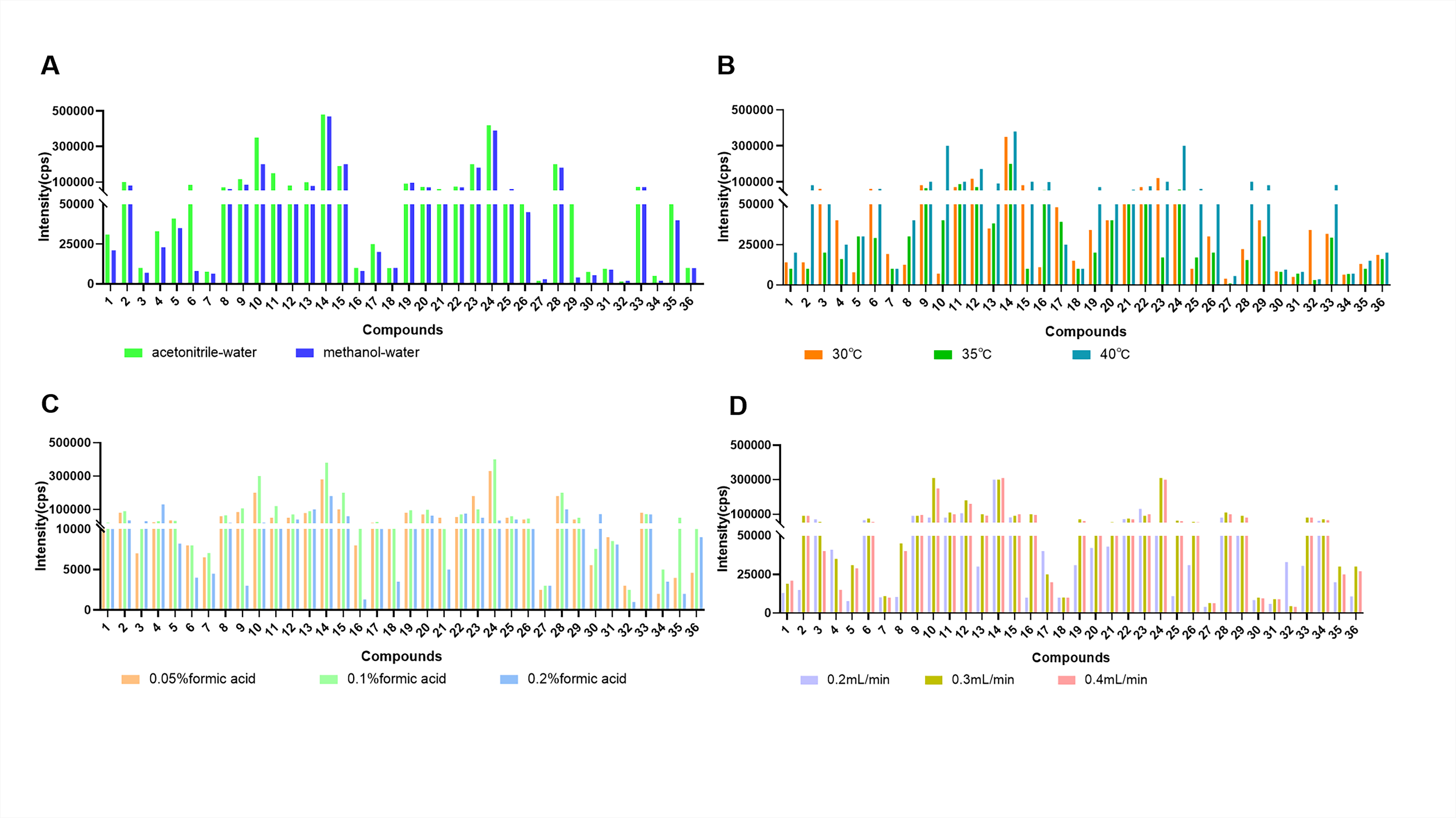

Supplement: Supplementary file 2 [file Image1.TIF]
