# Supplementary material for: Simultaneous determination of multiple components in rat plasma by UHPLC-sMRM for pharmacokinetic studies after oral administration of Qingjin Yiqi Granules
Source: Front Pharmacol. 2023 Apr 13;14:1155973. doi: 10.3389/fphar.2023.1155973 (PMC10133546; doi:10.3389/fphar.2023.1155973)
Supplement: Supplementary file 3 [file DataSheet1.docx]

Supplementary Material

Simultaneous determination of multiple components in rat plasma by UHPLC-sMRM for pharmacokinetic studies after oral administration of Qingjin Yiqi Granules

**Xiaohua Yang^1^_’_^2^, Shujing Chen^1^_’_^2^, Kunze Du^1^_’_^2^, Ye Shang^1^_’_^2^, Shiming Fang^1^, Jin Li^1^, Han Zhang^1,3*^, Yanxu Chang^1,2,3^***

^1^ State Key Laboratory of Component-based Chinese Medicine, Tianjin University of Traditional Chinese Medicine, Tianjin, 301617, China;

^2^Tianjin Key Laboratory of Phytochemistry and Pharmaceutical Analysis, Tianjin University of Traditional Chinese Medicine, Tianjin, 301617, China;

^3^ Haihe Laboratory of Modern Chinese Medicine, Tianjin 301617, China

*** Correspondence:**Yanxu Chang, State Key Laboratory of Component-based Chinese Medicine, Tianjin University of Traditional Chinese Medicine

E-mail: zhanghan0023@126.com (H. Zhang) and Tcmcyx@126.com (Y.Chang)

# Supplementary Figures and Tables

## Supplementary Figures

**Supplementary Fig. 1.** Effects of some factors in liquid chromatography conditions on intensity of 36 chemical components. (A) mobile phases; (B) column temperatures; (C) concentrations of additive; (D) flow rate. 1-36 represented the compounds that were displayed in Table 1.

**Supplementary Fig. 1.** Effects of some factors in pretreatment of biological samples on extraction recovery and matrix effect of 36 chemical components. (A) and (B) type of extraction solvent; (C) and (D) vortex time; (E) and (F) resolution solvent. 1-36 represented the compounds that were displayed in Table 1. The values of the two red reference lines are 85.0% and 115.0%.

## Supplementary Tables

**Supplementary Table 1.** The sMRM parameters for detecting of 33 compounds and 3 internal standards (ISs).

**Supplementary Table 2.** Regressive equations, correlation coefficient, linear range and LLOQ of 33 analytes from QJYQ.

**Supplementary Table 3.** The comparison of various methods.

**
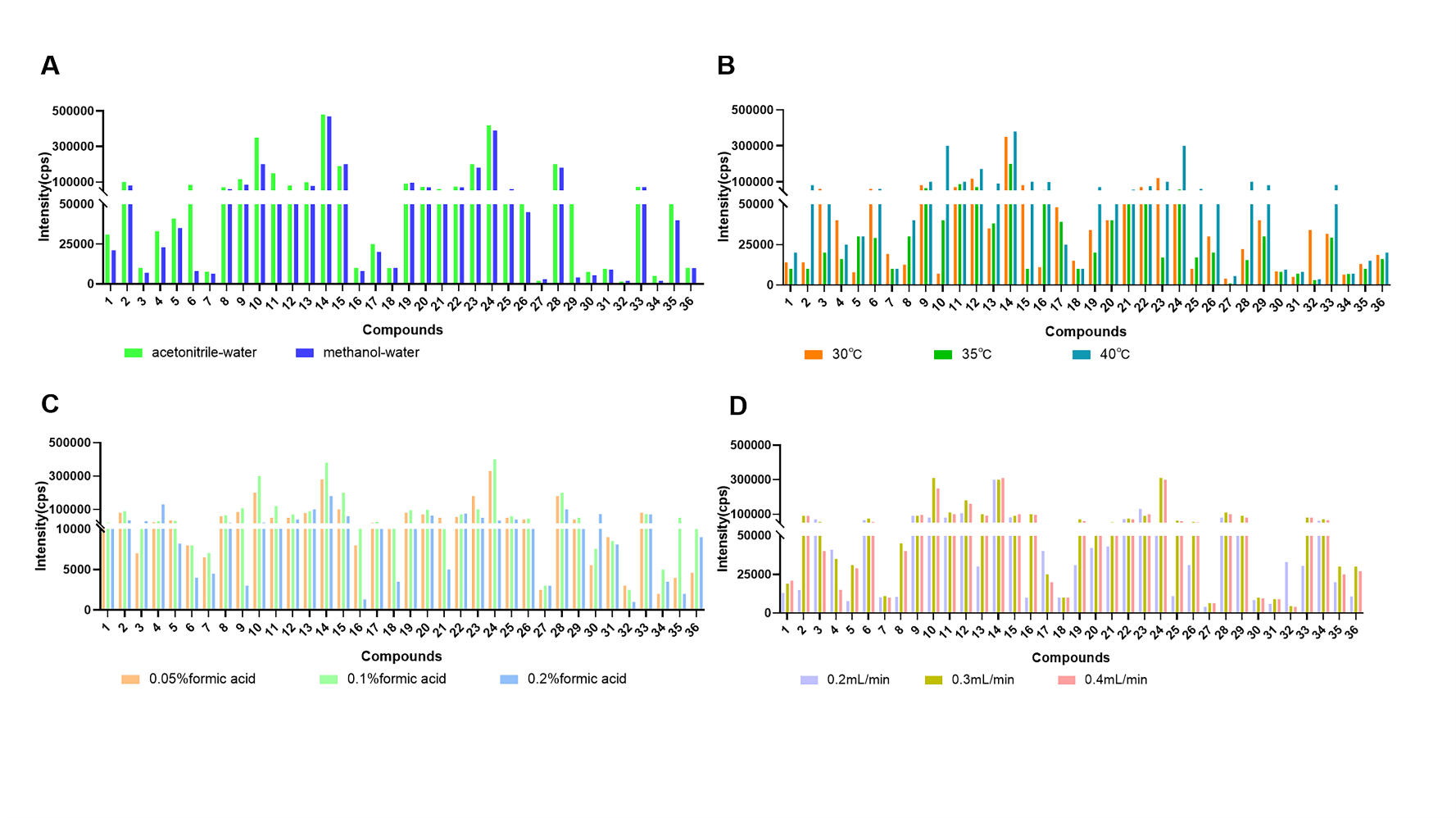
**

**Supplementary Fig. 1.** Effects of some factors in liquid chromatography conditions on intensity of 36 chemical components. (A) mobile phases; (B) column temperatures; (C) concentrations of additive; (D) flow rate. 1-36 represented the compounds that were displayed in Table 1.


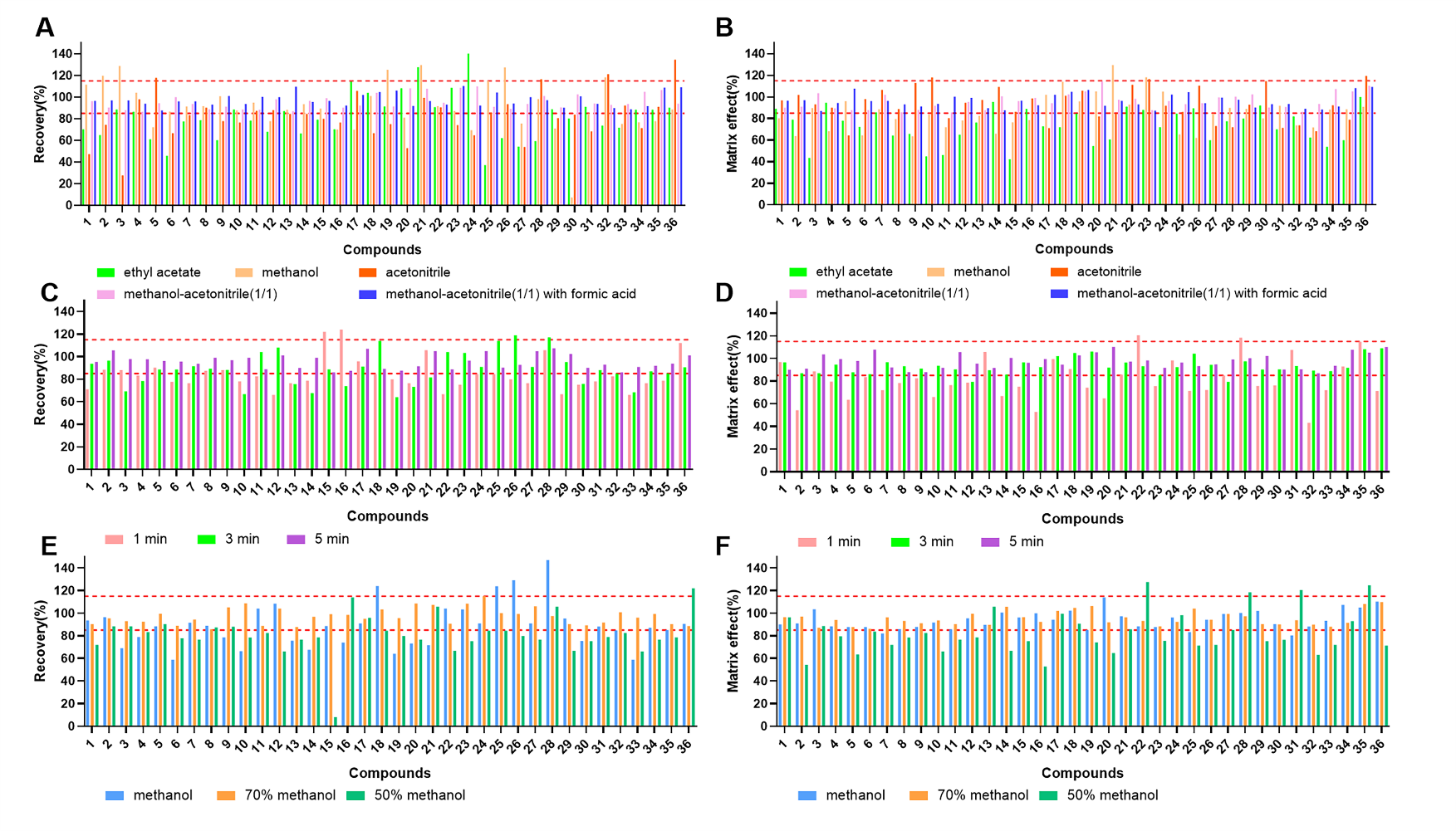


**Supplementary Fig. 1.** Effects of some factors in pretreatment of biological samples on extraction recovery and matrix effect of 36 chemical components. (A) and (B) type of extraction solvent; (C) and (D) vortex time; (E) and (F) resolution solvent. 1-36 represented the compounds that were displayed in Table 1. The values of the two red reference lines are 85.0% and 115.0%.

**Supplementary Table 1. The sMRM parameters for detecting of 33 compounds and 3 internal standards (ISs).**

| NO. | Compounds | Ion mode | RT (min) | Formula | Q1 | Q3 | DP(V) | EP(V) | CE(V) | CXP(V) |
| --- | --- | --- | --- | --- | --- | --- | --- | --- | --- | --- |
| 1 | harpagide | N | 2.10 | C_15_H_24_O_10_ | 362.9 | 138.9 | -79.9 | -12.1 | -30.1 | -12.2 |
| 2 | protocatechuic acid | N | 2.40 | C_7_H_6_O_4_ | 152.9 | 109.0 | -50.5 | -10.6 | -20.0 | -10.0 |
| 3 | atractyloside A | P | 3.90 | C_21_H_36_O_10_ | 466.4 | 251.0 | 70.1 | 10.2 | 15.6 | 13.6 |
| 4 | catechin | N | 4.37 | C_15_H_14_O_6_ | 289.1 | 245.0 | -103.4 | -6.2 | -20.2 | -12.1 |
| 5 | chlorogenic acid | N | 4.72 | C_16_H_18_O_9_ | 352.9 | 191.0 | -44.5 | -8.0 | -19.9 | -6.7 |
| 6 | cryptochlorogenic acid | N | 5.44 | C_16_H_18_O_9_ | 352.8 | 191.1 | -39.2 | -9.7 | -27.8 | -6.2 |
| 7 | verbenalin | N | 5.56 | C_17_H_24_O_10_ | 433.0 | 225.0 | -60.0 | -5.0 | -20.0 | -15.0 |
| 8 | paeoniflorin | N | 6.20 | C_23_H_28_O_11_ | 525.1 | 449.1 | -34.1 | -9.3 | -19.4 | -15.9 |
| 9 | *p*-Coumaric acid | N | 6.32 | C_9_H_8_O_3_ | 163.0 | 119.0 | -55.0 | -10.0 | -20.3 | -10.0 |
| 10 | ferulic acid | N | 7.08 | C_10_H_10_O_4_ | 192.9 | 134.0 | -49.3 | -12.1 | -19.6 | -33.9 |
| 11 | sinapic acid | N | 7.26 | C_11_H_12_O_5_ | 222.9 | 164.0 | -60.1 | -4.2 | -19.2 | -10.5 |
| 12 | vitexin | N | 7.30 | C_21_H_20_O_10_ | 431.0 | 311.0 | -120.0 | -12.7 | -30.2 | -10.6 |
| 13 | liquiritin | N | 7.32 | C_21_H_22_O_9_ | 417.0 | 134.8 | -92.2 | -10.9 | -39.3 | -33.4 |
| 14 | liquiritin apioside | N | 7.43 | C_26_H_30_O_13_ | 549.1 | 255.0 | -129.0 | -7.1 | -41.2 | -8.5 |
| 15 | isoferulic acid | N | 7.48 | C_10_H_10_O_4_ | 193.0 | 133.0 | -39.6 | -10.0 | -32.3 | -12.5 |
| 16 | hyperoside | N | 7.67 | C_21_H_20_O_12_ | 463.0 | 300.9 | -116.0 | -10.0 | -30.4 | -13.7 |
| 17 | cimifugin | P | 7.80 | C_16_H_18_O_6_ | 307.1 | 259.0 | 40.0 | 10.5 | 42.7 | 10.8 |
| 18 | scutellarin | N | 7.92 | C_21_H_18_O_12_ | 460.9 | 284.8 | -30.9 | -4.7 | -34.8 | -10.2 |
| 19 | quercitrin | N | 9.03 | C_21_H_20_O_11_ | 447.0 | 300.0 | -98.5 | -5.0 | -35.0 | -14.1 |
| 20 | hesperidin | N | 9.12 | C_28_H_34_O_15_ | 609.0 | 301.0 | -133.0 | -3.6 | -31.4 | -21.4 |
| 21 | apigenin-7-*O*-glucuronide | N | 9.25 | C_21_H_18_O_11_ | 445.1 | 268.8 | -87.9 | -14.3 | -31.0 | -13.9 |
| 22 | isoliquiritin apioside | N | 10.32 | C_26_H_30_O_13_ | 549.0 | 254.9 | -144.0 | -4.1 | -37.4 | -11.1 |
| 23 | ononin | N | 10.63 | C_22_H_22_O_9_ | 475.0 | 266.8 | -70.0 | -10.0 | -20.2 | -10.3 |
| 24 | baicalin | N | 10.90 | C_21_H_18_O_11_ | 445.0 | 268.9 | -64.1 | -4.8 | -29.8 | -21.9 |
| 25 | isoliquiritin | N | 11.02 | C_21_H_22_O_9_ | 417.1 | 254.9 | -130.0 | -12.2 | -25.8 | -13.3 |
| 26 | harpagoside | N | 13.00 | C_24_H_30_O_11_ | 493.0 | 147.0 | -66.7 | -7.1 | -23.8 | -40.2 |
| 27 | wogonoside | N | 13.62 | C_22_H_20_O_11_ | 459.0 | 268.0 | -179.0 | -11.0 | -32.0 | -30.0 |
| 28 | naringenin | N | 14.15 | C_15_H_12_O_5_ | 270.8 | 150.9 | -90.0 | -9.4 | -25.3 | -8.1 |
| 29 | baicalein | N | 15.84 | C_15_H_10_O_5_ | 268.8 | 195.0 | -122.0 | -12.2 | -38.1 | -12.6 |
| 30 | ginsenoside Rf | N | 15.97 | C_42_H_72_O_14_ | 799.2 | 637.3 | -211.0 | -5.1 | -42.5 | -14.3 |
| 31 | ginsenoside Rh1 | N | 16.83 | C_36_H_62_O_9_ | 637.3 | 475.3 | -139.0 | -5.0 | -30.4 | -13.7 |
| 32 | glycyrrhizic acid | N | 17.56 | C_42_H_62_O_16_ | 821.1 | 351.1 | -172.0 | -10.9 | -56.5 | -12.9 |
| 33 | wogonin | N | 17.91 | C_16_H_12_O_5_ | 282.8 | 267.9 | -77.9 | -4.5 | -23.4 | -10.8 |
| 34 | icariin (IS) | N | 14.42 | C_33_H_40_O_15_ | 721.3 | 513.0 | -70.2 | -10.0 | -25.1 | -10.8 |
| 35 | isopimpinellin (IS) | P | 16.11 | C_13_H_10_O_5_ | 247.2 | 217.3 | 80.2 | 6.1 | 10.2 | 6.3 |
| 36 | astragaloside II (IS) | N | 17.70 | C_43_H_70_O_15_ | 871.3 | 825.5 | -114.0 | -12.0 | -35.1 | -19.8 |

**Supplementary Table 2. Regressive equations, correlation coefficient, linear range and LLOQ of 33 analytes from QJYQ**

| NO. | Compounds | Regression equation | *r* | Linearity ranges (ng/mL) | LLOQ  (ng/mL) | Weight coefficient |
| --- | --- | --- | --- | --- | --- | --- |
| 1 | harpagide | Y=3.91E-005X-0.000397 | 0.9976 | 1.56-1000 | 1.06 | 1/X^2^ |
| 2 | protocatechuic acid | Y=0.00134X+0.0048 | 0.9928 | 1.56-1000 | 0.72 | 1/X^2^ |
| 3 | atractyloside A | Y=0.000482X+0.000997 | 0.9947 | 50.0-1000 | 14.0 | 1/X^2^ |
| 4 | catechin | Y=0.000491X+0.00117 | 0.9955 | 12.5-1000 | 7.69 | 1/X^2^ |
| 5 | chlorogenic acid | Y=0.00181X+0.00171 | 0.9952 | 12.5-1000 | 7.76 | 1/X^2^ |
| 6 | cryptochlorogenic acid | Y=0.000464X+0.00188 | 0.9951 | 12.5-1000 | 2.01 | 1/X |
| 7 | verbenalin | Y=0.000112X-0.00055 | 0.9969 | 12.5-1000 | 11.2 | 1/X^2^ |
| 8 | paeoniflorin | Y=0.00103X-2.64E-006 | 0.9973 | 12.5-1000 | 4.08 | 1/X^2^ |
| 9 | *p*-Coumaric acid | Y=0.00207X+0.0161 | 0.9940 | 1.56-1000 | 0.63 | 1/X^2^ |
| 10 | ferulic acid | Y=0.000751X+0.000438 | 0.9969 | 12.5-1000 | 4.50 | 1/X^2^ |
| 11 | sinapic acid | Y=0.00049X-0.00051 | 0.9937 | 1.56-1000 | 0.75 | 1/X^2^ |
| 12 | vitexin | Y=0.00359X-0.00115 | 0.9979 | 1.56-1000 | 1.43 | 1/X^2^ |
| 13 | liquiritin | Y=0.000697X+0.00102 | 0.9987 | 1.56-1000 | 1.50 | 1/X^2^ |
| 14 | liquiritin apioside | Y=0.000588X+0.000765 | 0.9987 | 12.5-1000 | 8.64 | 1/X^2^ |
| 15 | isoferulic acid | Y=3.49E-005X-0.000163 | 0.9961 | 50.0-1000 | 46.3 | 1/X^2^ |
| 16 | hyperoside | Y=0.0027X-0.000241 | 0.9945 | 1.56-1000 | 0.43 | 1/X^2^ |
| 17 | cimifugin | Y=0.00745X-0.000363 | 0.9944 | 1.56-1000 | 0.80 | 1/X^2^ |
| 18 | scutellarin | Y=0.000112X-0.000435 | 0.9983 | 50.0-1000 | 23.4 | 1/X^2^ |
| 19 | quercitrin | Y=0.00367X-0.000649 | 0.9963 | 1.56-1000 | 0.81 | 1/X^2^ |
| 20 | hesperidin | Y=2.63E-005X+0.000454 | 0.9965 | 12.5-1000 | 8.40 | 1/X^2^ |
| 21 | apigenin-7-*O*-glucuronide | Y=0.00124X-0.00019 | 0.9948 | 1.56-1000 | 1.26 | 1/X^2^ |
| 22 | isoliquiritin apioside | Y=0.00364X-0.000413 | 0.9936 | 1.56-1000 | 1.10 | 1/X^2^ |
| 23 | ononin | Y=0.002X-0.000416 | 0.9956 | 1.56-1000 | 1.36T | 1/X^2^ |
| 24 | baicalin | Y=0.000281X+0.00356 | 0.9983 | 15.6-10000 | 10.9 | 1/X^2^ |
| 25 | isoliquiritin | Y=0.00517X+0.00113 | 0.9965 | 1.56-1000 | 1.14 | 1/X^2^ |
| 26 | harpagoside | Y=0.000769X-2.87E-005 | 0.9935 | 1.56-1000 | 0.78 | 1/X^2^ |
| 27 | wogonoside | Y=5.94E-006X+2.12E-005 | 0.9936 | 125-10000 | 60.5 | 1/X |
| 28 | naringenin | Y=0.00262X+0.00124 | 0.9929 | 12.5-1000 | 2.20 | 1/X |
| 29 | baicalein | Y=0.000217X+0.00106 | 0.9930 | 12.5-1000 | 12.4 | 1/X^2^ |
| 30 | ginsenoside Rf | Y=0.000176X-0.00354 | 0.9930 | 12.5-1000 | 11.8 | 1/X^2^ |
| 31 | ginsenoside Rh1 | Y=0.000962X+0.00381 | 0.9952 | 50.0-1000 | 34.9 | 1/X^2^ |
| 32 | glycyrrhizic acid | Y=1.07E-005X+0.000342 | 0.9933 | 12.5-1000 | 12.5 | 1/X^2^ |
| 33 | wogonin | Y=0.00275X+0.00256 | 0.9987 | 1.56-1000 | 0.90 | 1/X^2^ |

**Supplementary Table 3. The comparison of various methods**

| Object | Num of analytes | Analysis method | Scan mode | Analysis time (min) | Injection volume(uL) | Liner range (ng/mL) | LLOQ (ng/mL) | References |
| --- | --- | --- | --- | --- | --- | --- | --- | --- |
| Fructus aurantia-Magnolia Bark Decoction | 7 | UHPLC-PDA | - | 25 | 3 | 100-14000 | - | (Xing, et al., 2013) |
| *Scutellariae Radix* | 6 | HPLC-MS/MS | MRM | 14 | 5 | 13.02-3333.33 | 1.3 | (Cai, et al., 2016) |
| *Rhizoma Cimicifugae* | 1 | HPLC-UV | - | 10 | 20 | 20.6–5000.15 | 20.6 | (Si, et al., 2008) |
| *Salvia miltiorrhiza Bge.* and *Carthamus tinctorius L.* | 4 | HPLC-DAD | - | 31 | 20 | 1000-200000 | 90.0 | (Zhao, et al., 2019) |
| *Acacia catechu (L.f.) Willd* and *Scutellaria baicalensis Georgi* | 4 | HPLC-MS/MS | MRM | 25 | 5 | 550-100000 | 10.0 | (Wang, et al., 2019) |
| Xuanmai Ganjie Granules | 5 | UHPLC-MS/MS | MRM | 8 | 5 | 1.5-30 | 1.5 | (Wu, et al., 2019) |
| Gan-Sui-Ban-Xia Decoction | 4 | UHPLC-MS/MS | MRM | 5.2 | 5 | 4.32-2210 | 1.4 | (Zhang, et al., 2015) |
| Dahuang-Gancao decoction | 7 | UHPLC-MS/MS | MRM | 14 | 2 | 2.265-4530 | 2.265 | (Chen, et al., 2019) |
| Shengjiang Xiexin decoction | 16 | UHPLC-MS/MS | MRM | 28 | 10 | 1-100 | 1.0 | (Guan, et al., 2017) |
| QJYQ | 33 | UHPLC-MS/MS | sMRM | 20 | 2 | 1.56-1000 | 0.4 | This work |

# Reference

Cai, Y., Li, S., Li, T., Zhou, R., Wai, A.T., & Yan, R. (2016). Oral pharmacokinetics of baicalin, wogonoside, oroxylin A 7-O-beta-d-glucuronide and their aglycones from an aqueous extract of Scutellariae Radix in the rat. *J Chromatogr B Analyt Technol Biomed Life Sci*, 1026, 124-133, doi:10.1016/j.jchromb.2015.11.049

Chang, Y.X., Ge, A.H., Yu, X.A., Jiao, X.C., Li, J., He, J., et al. (2016). Simultaneous determination of four phenolic acids and seven alkaloids in rat plasma after oral administration of traditional Chinese medicinal preparation Jinqi Jiangtang Tablet by LC-ESI-MS/MS. *J Pharm Biomed Anal*, 117, 1-10, doi:10.1016/j.jpba.2015.08.030

Chen, Y.Y., Cao, Y.J., Tang, Y.P., Yue, S.J., & Duan, J.A. (2019). Comparative pharmacodynamic, pharmacokinetic and tissue distribution of Dahuang-Gancao decoction in normal and experimental constipation mice. *Chin J Nat Med*, 17(11), 871-880, doi:10.1016/S1875-5364(19)30104-9

Guan, H., Wang, X., Wang, S., He, Y., Yue, J., Liao, S., et al. (2017). Comparative intestinal bacteria-associated pharmacokinetics of 16 components of Shengjiang Xiexin decoction between normal rats and rats with irinotecan hydrochloride (CPT-11)-induced gastrointestinal toxicity in vitro using salting-out sample preparation and LC-MS/MS. *RSC Advances*, 7(69), 43621-43635, doi:10.1039/c7ra03521g

Si, D., Sun, X., Qi, D., Chen, X., & Bi, K. (2008). Determination and pharmacokinetics of isoferulic acid in rat plasma by high-performance liquid chromatography after oral administration of isoferulic acid and Rhizoma Cimicifugae extract. *J Pharm Biomed Anal*, 47(1), 140-145, doi:10.1016/j.jpba.2007.12.044

Tong, L., Wan, M., Zhang, L., Zhu, Y., Sun, H., & Bi, K. (2012). Simultaneous determination of baicalin, wogonoside, baicalein, wogonin, oroxylin A and chrysin of Radix scutellariae extract in rat plasma by liquid chromatography tandem mass spectrometry. *J Pharm Biomed Anal*, 70, 6-12, doi:10.1016/j.jpba.2012.03.051

Wang, L., Shen, X., Mi, L., Jing, J., Gai, S., Liu, X., et al. (2019). Simultaneous determinations of four major bioactive components in Acacia catechu (L.f.) Willd and Scutellaria baicalensis Georgi extracts by LC-MS/MS: Application to its herb-herb interactions based on pharmacokinetic, tissue distribution and excretion studies in rats. *Phytomedicine*, 56, 64-73, doi:10.1016/j.phymed.2018.09.239

Wu, Y., Wang, P., Yang, H., & Sui, F. (2019). UPLC-Q-TOF-MS and UPLC-MS/MS methods for metabolism profiles and pharmacokinetics of major compounds in Xuanmai Ganjie Granules. *Biomed Chromatogr*, 33(3), e4449, doi:10.1002/bmc.4449

Xing, Z.h., Peng, W.J., Huang, W., Huang, X., & Liu, W.p. (2013). Analysis of Major Constituents in Fructus aurantii-Magnolia Bark Decoction by UPLC-PDA. *J. Chromatogr. Sci.* 52(8), 826-830, doi:10.1093/chromsci/bmt122

Zhang, Y., Qian, D., Pan, Y., Zhu, Z., Huang, J., Xi, J., et al. (2015). Comparisons of the pharmacokinetic profile of four bioactive components after oral administration of gan-sui-ban-xia decoction plus-minus gansui and gancao drug combination in normal rats. *Molecules*, 20(5), 9295-9308, doi:10.3390/molecules20059295.

Zhao, X., Yu, L., Chen, Y., Wang, Y., Wan, H., & Yang, J. (2019). Comparative Pharmacokinetics of Hydrophilic Components in Salvia miltiorrhiza Bge. and Carthamus tinctorius L. in Rats That Underwent Cerebral Ischemia Reperfusion Using an HPLC-DAD Method. *Front Pharmacol*, 10, 1598, doi:10.3389/fphar.2019.01598.
